# Effects of Astragalus membranaceus fiber on growth performance, nutrient digestibility, microbial composition, VFA production, gut pH, and immunity of weaned pigs

**DOI:** 10.1002/mbo3.712

**Published:** 2018-08-16

**Authors:** Dongsheng Che, Seidu Adams, Cai Wei, Qin Gui‐Xin, Emmanuel M. Atiba, Jiang Hailong

**Affiliations:** ^1^ College of Animal Science and Technology Jilin Agricultural University Changchun China; ^2^ Key Laboratory of Animal Production Product Quality and Security Ministry of Education Changchun China; ^3^ Jilin Provincial Key Laboratory of Animal Nutrition and Feed Science Changchun China

**Keywords:** 16SrDNA, Astragalus membranaceus, growth performance, immunity, microbiota, piglets

## Abstract

Astragalus membranaceus is an herbaceous perennial plant, growing to about 2 feet tall, with sprawling stems and alternate leaves about 12–24 leaflets. In total, 24 cross bred (Duroc × Landrace × Yorkshire) piglets weaned at 4 weeks with an average body weight of 10.84 ± 1.86 kg, were divided into four groups and randomly assigned to dietary treatments containing different AMSLF levels (0.00%, 2.50%, 5.00%, and 7.50%). The piglets in the control group (0.00% AMSLF) were fed basal diet and other treatment groups were fed basal diet in addition to 2.50%, 5.00%, and 7.50% pulverized AMSLF. The results indicated that supplementation with AMSLF significantly (*p *<* *0.05) decreased diarrheal incidence in piglets. There was significant difference between treatment in terms of ADFI, ADG and FCR. Both 5.00% and 7.50% treatments significantly increased growth performance. The digestibility of gross energy and dry matter increased (*p *>* *0.05) with increasing AMSLF level. The level of blood IL‐2 and TNF‐α were significantly affected by AMSLF supplementation with 7.50% AMSLF group having higher (*p *<* *0.05) IL‐2 and TNF‐α levels than the other treatment groups. The 16SrDNA sequencing results from the four treatments showed that the potentially active bacterial microbial population and diversity in pig cecum were dominated by the phyla *Bacteriodetes* and *Firmicutes* regardless of the AMSLF supplementation. The Shannon diversity, PD whole tree diversity indices and Chao analyses exhibited significant variability in species richness across the treatments. The principal coordinates analysis (PCoA) showed significant (*p *<* *0.1) differences between bacterial communities in all treatment groups. Results from the current study suggested that AMSLF supplementation increased composition of bacterial microbiota in pig gut. In conclusion, dietary supplements with AMSLF could potentially be used to prevent diarrheal incidence and improved pig production.

## 1 INTRODUCTION

The emergence of antibiotic resistance among many pathogenic microorganisms and consumer demand for organic food production has necessitated the search for alternative approaches to control microbial infection in pigs. The application of plant remedies, particularly, Astragalus species seem to be one of the promising alternatives (Griggs & Jacob, 2005). Astragalus membranaceus is a leguminous plant from the two main varieties; Astragalus membranaceus (Fisch) Bunge variety mongholicus (Bunge) Hsiao and Astragalus membranaceus (Fisch) Bunge (Wang et al., 2016). Dried Astragalus roots contain chemical compounds such as alkaloids, polysaccharides, glycosides, flavonoids, amino acids, saponins, and trace mineral which exhibit both medicinal and nutritional characteristics (Xi et al., 2014). Astragalus membranaceus roots have been commonly used in Chinese Traditional Medicine for over 2,000 years to cure many diseases, including indigestion and it has been characterized with antimicrobial activity (Li et al., 2010; Qin et al., 2012; Shao et al., 2004; Xi et al., 2014). However, after harvesting the roots of Astragalus plant, the aerial parts which comprise of stems and leaves are discarded as waste posing a serious environmental management problem. Therefore, there is a need to explore the use of this waste as an alternative to antibiotic, at the same time minimize the risk of environmental problem. Livestock species, both ruminants and monogastric animals, particularly pig can play important role in managing Astragalus waste due to their ability to digest fibrous and by‐products, such materials may reduce production cost. However, early weaning pigs have great chances of growth depress and incidence of the gut disorder especially diarrhea mainly due to an immature gastrointestinal tract. In this study, we hypothesized that AMSLF may counteract the physiological and immunological stress conditions and improve growth performance of weaning piglets. Thus, this paper aims to report the effects of Astragalus membranaceus fiber on diarrheal incidence, performances, ceca microbial population, pH, and volatile fatty acids (VFA) in early weaning piglets.

## 2 MATERIALS AND METHODS

### 2.1 Animal care and ethics

This study was carried out in accordance with Jilin Agricultural University animal welfare and research ethics and provincial pig rules and regulations.

### 2.2 Astragalus membranaceus stems and leaves collection and preparation

The Astragalus membranaceus stems and leaves were collected from Department of Herbal Plants and Chinese Medicine Plantation Farm, Jilin Agricultural University. After drying AMSLF overnight at 60°C and pulversing it through a 5 mm sieve, the extract was stored in a closed vessel at room temperature prior to being incorporated into the experimental diets.

### 2.3 Experimental animals, design and management

In total, 24 cross bred (Duroc × Landrace × Yorkshire) piglets of 28 days old with an average body weight of 10.84 ± 1.86 kg was used in this study. The piglets were divided into four experimental groups with each group comprised of six piglets and arranged in a complete randomize block design. The animals were housed in pens (1.8 × 1.2 m) with plastic slatted floor, each pen contained two piglets. The pens were cleaned on a daily basis to prevent disease outbreak in piglets. The animals were allowed to acclimatize to the diets for a week prior to the beginning of the experiment. Four experimental diets containing 0.00% AMSLF, 2.50% AMSLF, 5.00% AMSLF and 7.50% AMSLF were formulated according to the standard of National Research Council (1998). The diets contained soybean meal and cracked corn as major sources of protein and energy respectively (Table 1). The piglets were fed twice a day at 05: 30 am and 05:30 pm and had free access to clean drinking water ad libitum. Feed intake and orts were recorded daily in the morning for each animal.

**Table 1:** Composition and nutrient levels of basal diets (%DM, unless otherwise noted)

Items	0.00% AMSLF	2.50% AMSLF	5.00% AMSLF	7.50% AMSLF
Cracked corn	61.53	59.5	56.79	54.30
Soybean meal	20.90	18.63	14.40	14.63
Fish meal	3.66	3.66	3.46	2.30
Whey powder	3.41	3.41	4.35	4.35
Fermented soybean meal	6.50	8.20	12.00	12.92
AMSLF	0.00	2.50	5.00	7.50
Premix	4.00	4.00	4.00	4.00
Calculated Nutritional levels				
ME (MJ·kg^−1^)	10.39	10.27	10.36	10.13
Crude protein %	19.91	19.93	19.86	19.89
Methionine %	0.36	0.35	0.34	0.32
Lysine %	1.24	1.22	1.21	1.18
Calcium %	0.82	0.81	0.81	0.80
Available phosphorus %	0.41	0.39	0.36	0.38

***Note.*** The premix provides the following per kilogram: vitamin A (KIU) 130–396, Kilo‐vitamin D (KIU) 30–124, Vitamin E 400, Vitamin K2 40–150) 25, Vitamin B2 75–1,500 mg 4,500–1,500 mg Iron 1,500–3,700 Magnesium 400–3,700 Moisture 9% Sodium (%) 6–14, Total Phosphorus 2.0, Lysine 1.3, Calcium 10–20, Phytase U 12,500. 0.00% AMSLF: No added quantity of Astragalus membranaceus stems and leaves fiber; 2.50% AMSLF: 2.50% of Astragalus membranaceus stems and leaves fiber added to a quantity of the diet; 5.00% AMSLF: 5.00% of Astragalus membranaceus stems leaves fiber added to a quantity of the diet; 7.50% AMSLF of Astragalus membranaceus stems and leaves fiber added to the quantity of the diet.

### 2.4 Diarrheal incidence

The piglets were monitored daily for diarrheal signs, those that had watery feces were classified either pasty or fluid and recorded as diarrheal case. The diarrheal occurrence (%) was estimated as described by Hu et al. (2014).

### 2.5 Growth performance, feed conversion ratio, and nutrient digestibility

The piglets were weighed early in the morning prior to feeding on the first and last days of feeding trials. The initial body weight was subtracted from the final body weight and divided by the days of experiment to obtain average daily gain for each animal. Average feed intake (ADFI) was determined by calculating total feed consumed per day. Feed conversion ratio (FCR) was determined by dividing the feed intake by the body weight gain for each piglet. On the 25th, 26th, and 27th of the experiment, 100 g of fresh excrement was collected directly from the rectum of each pig. The fecal samples were homogenized and stored at −20°C until laboratory analysis was done. The samples were analyzed for crude protein (CP), crude fiber (CF), gross energy (GE), and dry matter (DM) digestibility according to the procedures described by Hassanat, Gervais, and Benchaar (2017).

### 2.6 Immunological parameters, cecum pH, and volatile fatty acids

At the end of the experiment, three pigs were randomly selected from each experimental group and slaughtered. Blood samples were collected and analyzed for serum antibodies of TNF‐α and IL‐2, IgA, IgG, and IgM using pig ELISA TNF‐α, Pierce Endogen and IL‐2 ELISA Kit, No. ABIN365284, UK respectively. Cecum digest was collected from each animal and pH was measured using the portable pH meter. About 100 g of the digest samples was taken and frozen at −20°C for VFA analysis. Three major VFAs were analyzed according to the methods described by Freire, Guerreiro, Cunha, and Aumaitre (2000). Another 10 g of cecum content was sampled from each animal and transported to the laboratory for microbial analysis.

### 2.7 Total DNA extraction and PCR amplification

Extraction of total DNA was performed as described by Corrigan, de Leeuw, Penaud‐Frézet, Dimova, and Murphy (2015) using QIAamp DNA Mini Stool Kit (QIAGEN, MD, USA). Briefly, a sample of cecum content was ground to a fine powder using a mortar and pestle, combined with proteinase, transferred to centrifuge tube and incubated for 40 min at 50–55°C water bath to thaw. The samples were centrifuged at 10,000*g* for 10 min and the supernatant was removed and combined with preheated 2% agarose mixture followed by washing in 10% volume of TE buffer. The genomic DNA was quantified and quality checked using Nano drop 2000C spectrophotometer (Thermo Fisher Scientific Inc. Massachusetts, USA).

### 2.8 16SrDNA amplification and full‐length V3–4 region sequencing

The extracted DNA from cecum content samples was amplified using two sets of bacterial 341F (5‐CCTACACGACGCTCTTCCGATCTN‐3) and 805R (5‐GACTGGAGTTCCTTGGCACCCGAGAATTCCA‐3) according to methods described by Logares et al. (2013). The hypervariable V3–V4 regions of bacteria 16SrDNA were amplified by PCR at 95°C for 5 min denaturation, followed by 25 cycles at 95°C for 30 s, 55°C for 30 s, and 72°C for 30 s with extension at 72°C for 5 min. The PCR was performed in a mixture containing reagents and 50 ng of template DNA according to the procedure described by Drumo et al. (2016). The amplicons were extracted using 2% agarose gel electrophoresis and DNA was recovered using the agarose recovery kits. The PCR amplified product size were selected using Qubit 2.0 DNA assay kit and pooled in equimolar concentration and paired‐end sequenced on the Illumina platform (Hiseq or Miseq). Pair end reads were demultiplexed and quality filtered using the following control standard criteria: I. The 250 bp reads were removed from any site receiving an average quality score less than 20. II. Exact barcode matching, two nucleotide mismatch and reads with ambiguous characters were removed III. Reads that ranged between 220 and 500 nt were assembled according to their overlap sequence.

### 2.9 Operational taxonomic units (OTUs) picking and phylogenic‐diversity analysis

The OTUs were clustered with 97% similarity cut‐off using USEARCH and Chimeric sequences, subsequently filtered out to obtain OTUs for species classification (Edgar, 2013). Each sequence was randomly analyzed to avoid bias due to sample size differences. The DUDIPCA function was used to perform principal component analysis (PCoA) to determine status of the treatments. The most abundant sequences in each OTU were selected as a representative sequence then aligned against the core set of green genes in 16S database (http://greengenes.lbl.gov). After classification, the OTU index table was obtained according to the number of sequences in each OTU (McDonald et al., 2011).

### 2.10 Formation of prognostic functional profiles

The sequence number in each OTU was arranged in heat map using the R language g‐plot package in which the color gradient reflects the abundance of species in the different sample cluster. The sample alpha diversity index was determined using QIME software version 1.7 to create corresponding dilution curves based on the relative proportion of OTUs to the 16SrDNA sequence (Kemp & Aller, 2004). To ensure the comparability of the species between the samples, beta diversity index was used to analyze sample species complexity. Linear discrimination analysis (LDA) was also used to estimate the effect of the treatments on species abundances.

### 2.11 Statistical analysis

The data were analyzed using a one‐way analysis of variance ANOVA procedure of SPSS software version 20.0 (SPSS Inc., Chicago, IL, USA). A probability value of *p* valve <0.05 was considered statistically significant and where differences between means were noticeable, Duncan multiple range test (DMRT) was employed to ascertain the difference among the treatments.

## 3 RESULTS

### 3.1 Diarrheal incidence

Our results showed that supplementation with AMSLF significantly (*p *<* *0.05) decreased diarrheal incidence in piglets throughout the experimental period. The highest diarrhea occurrences were observed in the control group, whereas; the lowest diarrheal incidence was recorded in the 7.50% AMSLF group (Table 2).

**Table 2:** Diarrheal occurrence of weaned piglets fed diet supplemented with AMSLF (%)

Indicator	0.00% AMSLF	2.50% AMSLF	5.00% AMSLF	7.50% AMSLF	*p‐value*
Diarrheal incidence (%)	3.14 ± 0.351^a^	2.13 ± 0.36^b^	1.03 ± 0.36^c^	0.13 ± 0.35^d^	<0.01

***Note***. AMSLF means *Astragalus membranaceus* stem and leaves fiber. 0.00% AMSLF: No added quantity of Astragalus membranaceus stems and leaves fiber; 2.50% AMSLF: 2.50% of Astragalus membranaceus stems and leaves fiber added to a quantity of the diet; 5.00% AMSLF: 5.00% of Astragalus membranaceus stems leaves fiber added to a quantity of the diet; 7.50% AMSLF of Astragalus membranaceus stems and leaves fiber added to the quantity of the diet.

a,b,c,d Values within a row with different superscripts differ significantly at *p *<* *0.05.

### 3.2 Growth performance

The ADFI in the 5.00% AMSLF and the 7.50% AMSLF groups were higher (*p *<* *0.05) than in the 2.50% AMSLF and control groups. There was no difference between 2.50% AMSLF group and control group. However, ADG in the 2.50% AMSLF group was slightly higher than in the 5.00% AMSLF group. The FCR of piglets on 5.00% AMSLF and 7.50% AMSLF diets was higher (*p *<* *0.05) than those on 2.50% AMSLF and the control diets. The FCR of piglets in the 2.50% AMSLF group did not differ from that in the control group (Table 3).

**Table 3:** Influence of dietary AMSLF inclusion on growth performance of weaning pigs (g/day)

Indicators	0.00% AMSLF	2.50% AMSLF	5.00% AMSLF	7.50% AMSLF
ADFI	655.03 ± 12.73^a^	656.55 ± 13.11^a^	700.80 ± 10.09^b^	731.13 ± 18.60^b^
ADG	387.35 ± 16.51	391.60 ± 7.96	376.94 ± 10.59	377.78 ± 10.84
FCR	1.690 ± 0.009^a^	1.680 ± 0.016^a^	1.870 ± 0.049^b^	1.940 ± 0.088^b^

***Note.*** ADFI: Average daily feed intake; ADG: Average daily gain; FCR: Feed conversion ratio; AMSLF: Astragalus membranaceus stem and leaves fiber. 0.00% AMSLF: No added quantity of Astragalus membranaceus stems and leaves fiber; 2.50% AMSLF: 2.50% of Astragalus membranaceus stems and leaves fiber added to a quantity of the diet; 5.00% AMSLF: 5.00% of Astragalus membranaceus stems leaves fiber added to a quantity of the diet; 7.50% AMSLF of Astragalus membranaceus stems and leaves fiber added to the quantity of the diet.

a,b Values within a row with different superscripts differ significantly at *p *<* *0.05.

### 3.3 Nutrient digestibility

Our results showed that digestibility of DM, GE, CP, and CF were affected by AMSLF supplementation. The digestibility of DM, GE, CP, and CF in 7.50% AMSLF group was lower (*p *<* *0.05) than in 2.50% AMSLF group and the control groups but no difference was observed between 5.00% AMSLF and 7.50% AMSLF groups. The DM, GE, CP, and CF digestibility were higher in the 2.50% AMSLF group than in other treatment groups (Table 4).

**Table 4:** Influence of dietary treatments on nutrient digestibility on weaned pigs (% DM)

Indicators	0.00% AMSLF	2.50% AMSLF	5.00% AMSLF	7.50% AMSLF
CP	85.23 ± 1.69^ab^	84.26 ± 0.64^a^	82.22 ± 0.53^bc^	79.09 ± 0.67^c^
CF	59.02 ± 1.97^a^	56.42 ± 2.53^a^	39.05 ± 1.09^b^	34.42 ± 1.37^b^
GE	85.94 ± 0.82^a^	86.83 ± 0.82^a^	84.91 ± 0.32^a^	82.67 ± 0.15^b^
DM	85.89 ± 0.67^a^	87.08 ± 0.67^ab^	84.95 ± 0.22^b^	83.21 ± 0.18^c^

***Note*** **.** CP: Crude protein; CF: Crude fiber; GE: Gross Energy; DM: Dry matter; AMSLF means Astragalus membranaceus stem and leaves fiber. 0.00% AMSLF: No added quantity of Astragalus membranaceus stems and leaves fiber; 2.50% AMSLF: 2.50% of Astragalus membranaceus stems and leaves fiber added to a quantity of the diet; 5.00% AMSLF: 5.00% of Astragalus membranaceus stems leaves fiber added to a quantity of the diet; 7.50% AMSLF of Astragalus membranaceus stems and leaves fiber added to the quantity of the diet.

a,b,c Values within a row with different superscripts differ significantly at *p *<* *0.05.

### 3.4 Immunity

The level of blood IL‐2 and TNF‐α were significant affected by AMSLF supplementation. The 7.50% AMSLF group had higher (*p *<* *0.05) IL‐2 and TNF‐α levels than the other treatment groups (Table 5). No difference was observed in IgA, IgG, and IgM among the treated groups. However, IgA and IgM levels in AMSLF groups tended to increase slightly at the beginning then dropped gradually towards the end of the experiment.

**Table 5:** Influence of dietary AMSLF treatments on weaned piglets’ immunity (micrograms per millilitre)

Indicators	0.00% AMSLF	2.50% AMSLF	5.00% AMSLF	7.50% AMSLF
IL‐2	731.04 ± 39.57^a^	704.90 ± 20.12^a^	1035.78 ± 162.95^a^ ^c^	1352.86 ± 335.05^bc^
TNF‐α.	266.88 ± 31.20^bc^	222.58 ± 4.05^ac^	177.38 ± 24.72^a^	295.64 ± 14.12^b^
IgA	170.24 ± 7.04	173.23 ± 1.38	185.71 ± 4.02	179.65 ± 15.02
IgG	7.76 ± 0.29^a^	8.11 ± 0.31^a^	9.70 ± 0.14^b^	7.51 ± 0.07^a^
IgM	12.83 ± 2.36	15.42 ± 1.10	17.89 ± 4.34	15.07 ± 1.66

***Note.*** IL‐2: Interleukin‐2; TNF‐α: Tumor necrosis factor‐α; IgA: Immunoglobulins A; IgG: Immunoglobulins G; IgM: Immunoglobulins M; AMSLF means Astragalus membranaceus stem and leaves fiber. 0.00% AMSLF: No added quantity of Astragalus membranaceus stems and leaves fiber; 2.50% AMSLF: 2.50% of Astragalus membranaceus stems and leaves fiber added to a quantity of the diet; 5.00% AMSLF: 5.00% of Astragalus membranaceus stems leaves fiber added to a quantity of the diet; 7.50% AMSLF of Astragalus membranaceus stems and leaves fiber added to the quantity of the diet.

a,b,cValues within a row with different superscripts differ significantly at *p *<* *0.05.

### 3.5 pH and VFA concentration

The pH in the cecum contents did not differ between the treatment groups (Table 6). The AMSLF supplementation did not affect the concentration of acetic acid, butyric acid, and propionic acid in cecum content. Though, concentration of VFAs in the 2.50% AMSLF group seemed to be slightly greater than in the 5.00% AMSLF and the 7.50% AMSLF groups but the difference was not statistically significant.

**Table 6:** Effect of dietary AMSLF on pH and VFA (mmol/L) level in the ceca of weaned piglets

Indicators	0.00% AMSLF	2.50% AMSLF	5.00% AMSLF	7.50% AMSLF
Acetic acid	44.37 ± 2.04	53.55 ± 3.90	41.18 ± 3.08	40.65 ± 7.55
Propionic acid	24.12 ± 4.33	28.65 ± 3.89	25.07 ± 0.27	21.52 ± 4.99
Butyric acid	12.35 ± 1.69	17.03 ± 4.23	9.34 ± 0.29	10.92 ± 1.64
Total VFA	80.84 ± 4.25	99.23 ± 9.92	75.59 ± 2.58	73.09 ± 12.11
pH	5.55 ± 0.11	5.47 ± 0.08	5.66 ± 0.12	5.50 ± 0.03

***Note.*** AMSLF means Astragalus membranaceus stem and leaves fiber. 0.00% AMSLF: No added quantity of Astragalus membranaceus stems and leaves fiber; 2.50% AMSLF: 2.50% of Astragalus membranaceus stems and leaves fiber added to a quantity of the diet; 5.00% AMSLF: 5.00% of Astragalus membranaceus stems leaves fiber added to a quantity of the diet; 7.50% AMSLF of Astragalus membranaceus stems and leaves fiber added to the quantity of the diet.

### 3.6 Ceca microbial composition

**Figure 1:**
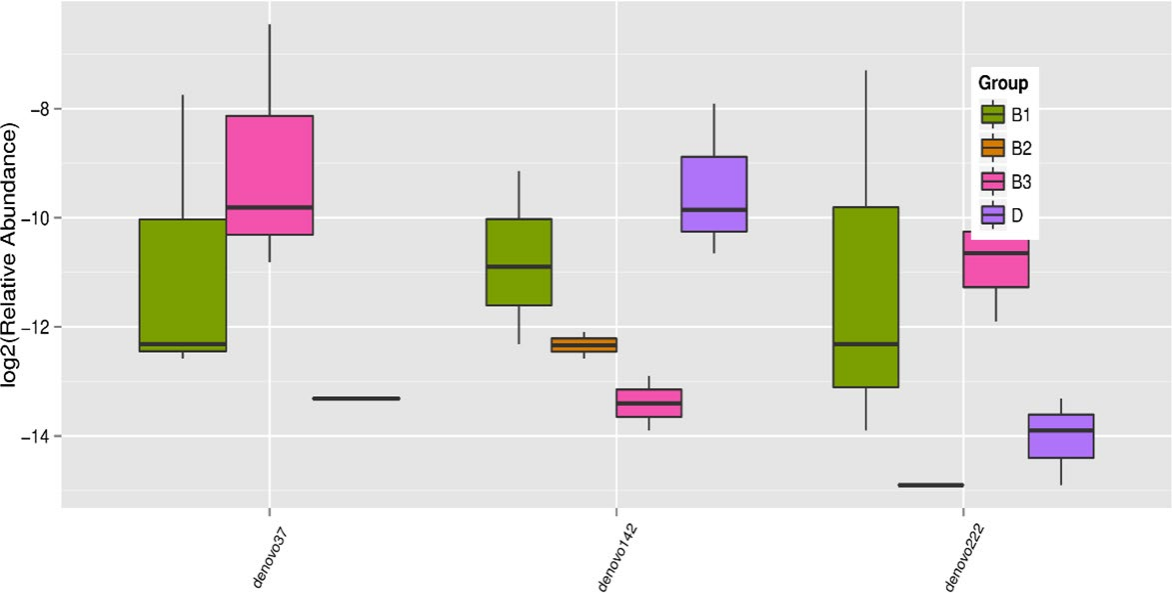
Shows the significant different (*p *<* *0.05) between the different treatment groups at the OTUs reads. The colors represent the groups and the OTUs represent each organism. Group B1: 2.50% AMSLF; Group B2: 5.00% AMSLF; Group B3: 7.50% AMSLF; Group D: 0.00% AMSLF

**Figure 2:**
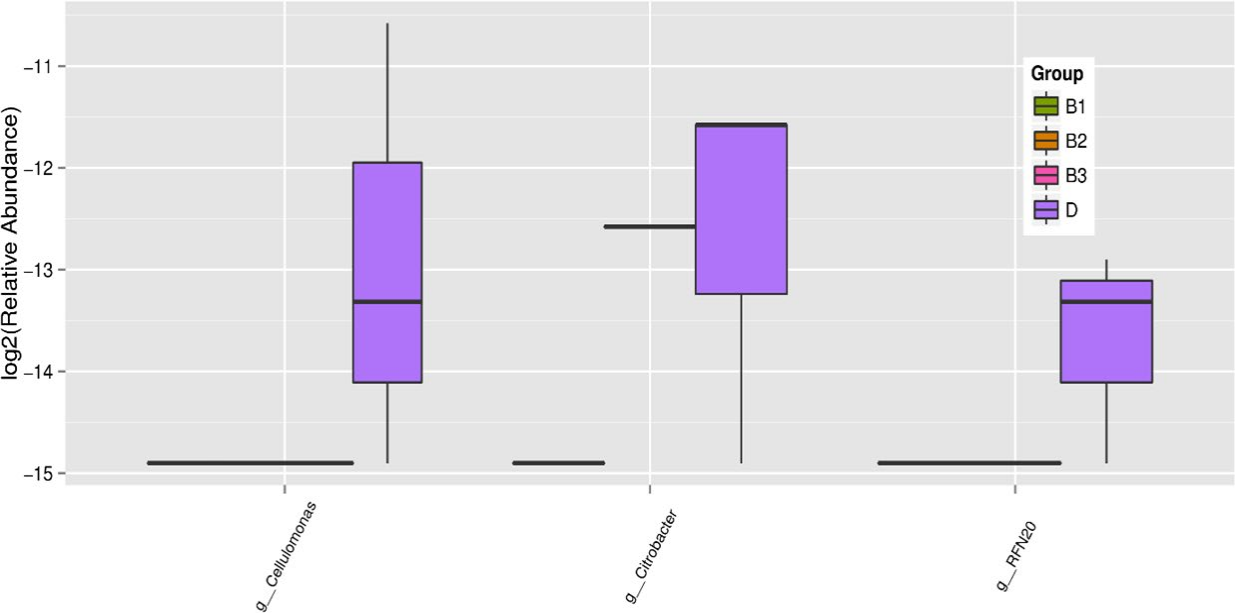
Shows the significant different in relative abundance (*p *<* *0.1) between the treatment groups and the control at the genus level. At the genus level, different colors represent the treatments. The relative abundance of g__Cellulomonas, g__Citrobacter, and g__RFN20 was significantly higher in the control group (D) in comparison with the treatment groups. Group B1: 2.50% AMSLF; Group B2: 5.00% AMSLF; Group B3: 7.50% AMSLF; Group D: 0.00% AMSLF

**Figure 3:**
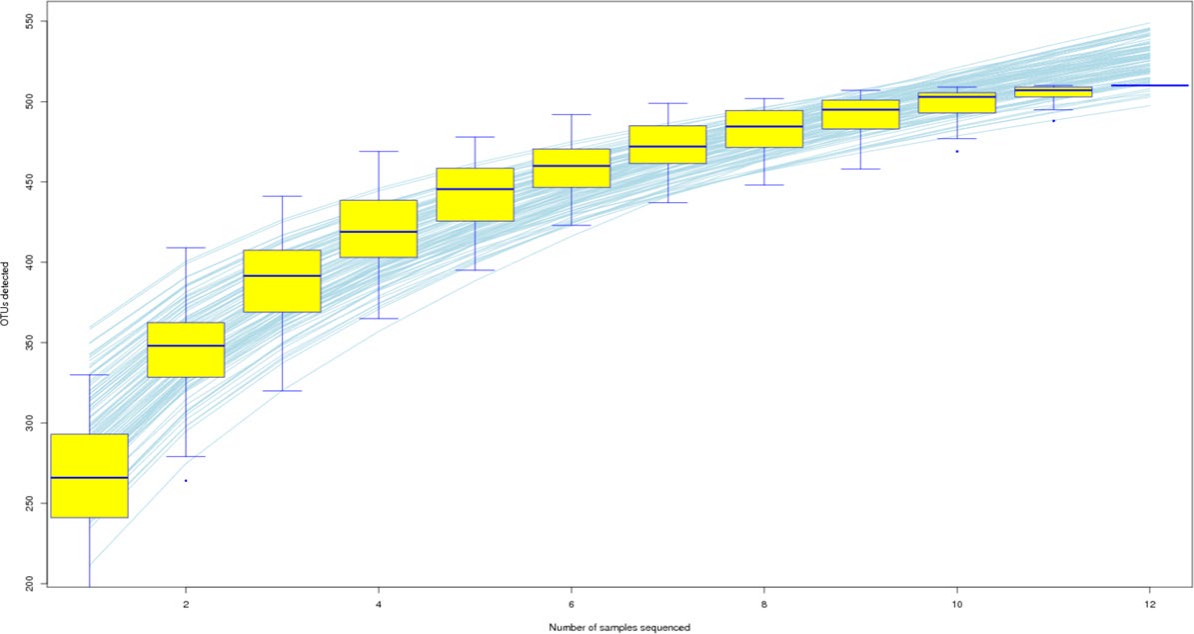
The rarefaction curves produced by the boxplots represent the number of sample sequence against the number of OTUs, and tended toward the saturation plateau

**Figure 4:**
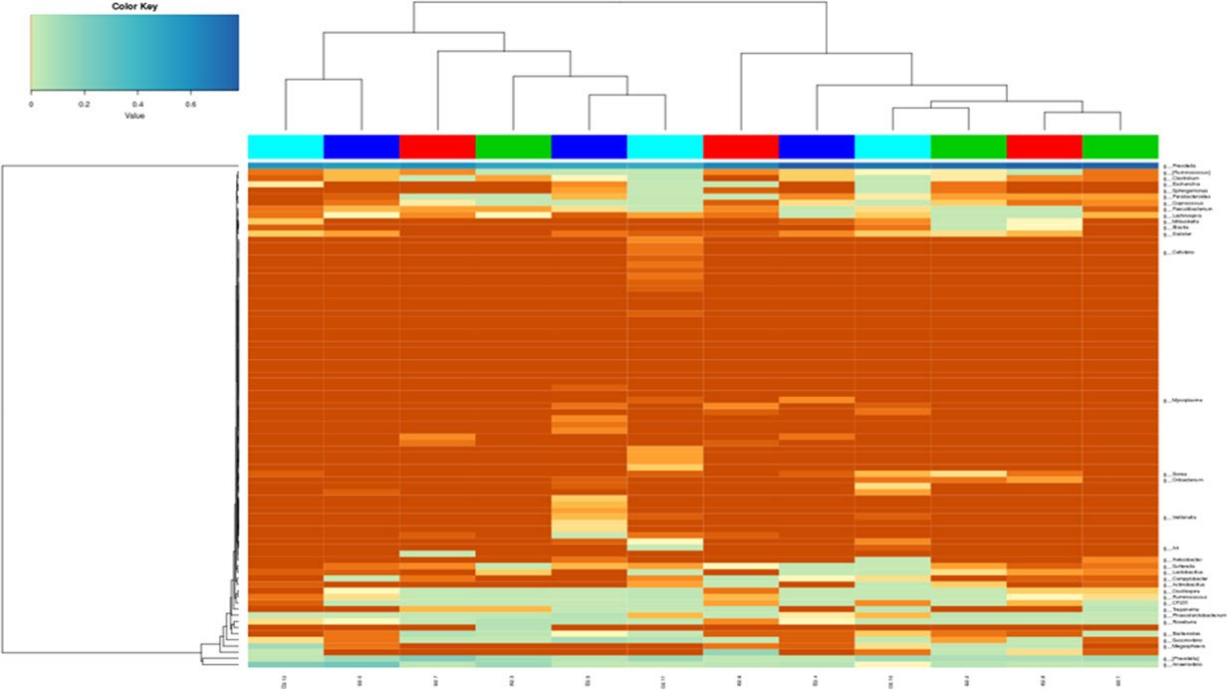
Shows the species abundance heatmap. Heatmap represents the size of the data matrix and allows clustering based on species or sample abundance similarity. The first two rows in the figure are the sample grouping information while, the color corresponds to the picture column. Group B1: 2.50% AMSLF; Group B2: 5.00% AMSLF; Group B3: 7.50% AMSLF; Group D: 0.00% AMSLF

**Figure 5:**
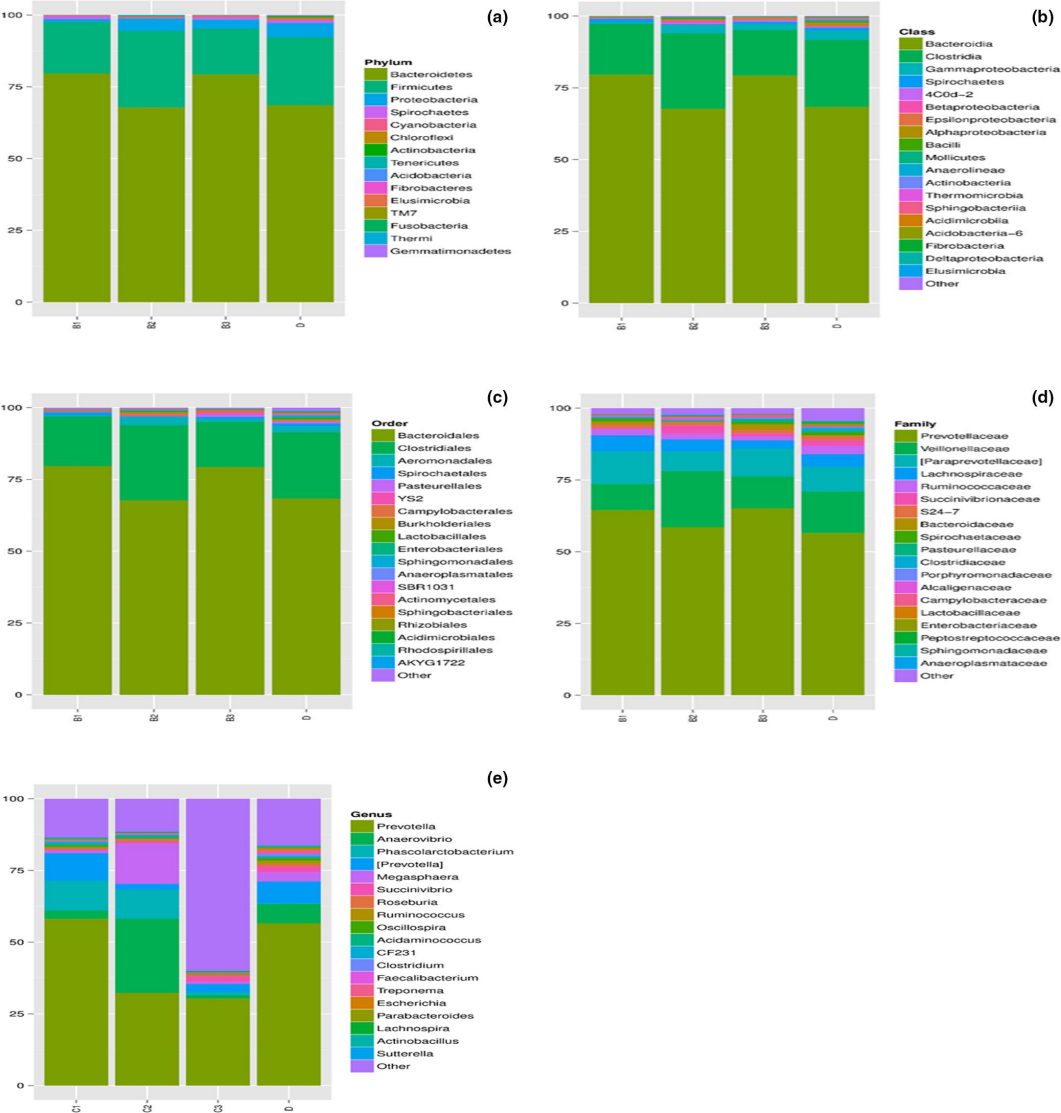
(a–e) Represented the microbial composition and diversity within the taxa. Group B1: 2.50% AMSLF; Group B2: 5.00% AMSLF; Group B3: 7.50% AMSLF; Group D: 0.00% AMSLF

The 16SrDNA gene pyrosequencing generated a total of 899292 quality sequence by an average of 261.67 per sample. The number of OTUs obtained was 510 based on the nucleotide sequence identity between the reads (Table 7). The minimum and maximum numbers of OTUs obtained per sample were 186 and 330 respectively. Moreover, significant differences between bacterial communities at OTUs level and genus level were noted in all the treatment groups (Figures 1 and 2). Rarefaction curves with levels tending to the plateau were generated to assess whether the samples provide sufficient OTU coverage (Figure 3). The Shannon diversity, PD whole tree diversity indices and Chao value for species richness were higher in the 2.50% AMSLF group than the other treatment groups (Supporting Information Figure S1). The Rarefaction analysis indicated good sequencing results, implying that our samples were sufficient to estimate bacterial species in the ceca microbiota of the piglets. The results of sample clustering showed the similarities and differences between the treatment groups. The beta diversity index microbiota composition of the piglets in different treatment groups was analyzed and visualized through PCoA analysis using weighted and unweighted UniFrac distance. Differences (*p *<* *0.1) between bacterial communities were observed in all treatment groups (Supporting Information Figure S2). A total of 510 phyla was detected in all the samples. Among which, *Bacteroidetes* was the most dominant phylum regardless of treatment, but their proportion varied considerably among the treatment groups. At the phylum level, different bacterial phyla were identified in all AMSLF groups. *Bacteroidetes, Firmicutes,* and *Proteobacteria* were detected as the dominant bacterial phyla, which made up 95% of the bacterial microbiota across the treatments (Supporting Information Figure S1; Figure 4). By comparison, phyla *Spirochaetes, Cyanobacteria,* and *Actinobacteria* were the less predominant and their total proportion in all treatment samples was 3.5% (Figure 5a).

**Table 7:** The taxonomic assignment of different OTUs

Name	Number of OTUs assigned
Phylum	510
Class	510
Family	339
Order	466
Genus	249
Species	36
Minimum number per sample	186
Maximum number per sample	330
Mean per sample	261.67
Standard deviation per sample	36.85

***Note.*** OTUs: Operational Taxonomic Units: In total, 510 OTUs were sequenced and assigned to different taxa. The minimum and maximum number of OTUs per sample was 186 and 330 respectively. The mean and standard deviation per sample was 261.67 and 36.85 respectively.

At the Class level, a total of 510 classes was observed across the treatments. Our results showed that *Bacteroidia*,* Clostridia*, and *Gammaproteobacteria* were the dominant classes in the samples. The relative abundance of *Bacteroidia* was significant higher (*p *<* *0.05) in the 2.50% AMSLF group and the 7.50% AMSLF group than in other groups, whereas, *Clostridia* was lower (*p *<* *0.05) in the 2.50% AMSLF and the 7.50% AMSLF groups. The abundance of *Gammaproteobacteria* was significantly lower at the 2.50% AMSLF group than in other groups, while *Betaproteobacteria* was significantly higher in the 5.00% AMSLF group and the 7.50% AMSLF group than in other groups (Figure 5b). At the family level, a total of 399 families was detected across the treatment samples. Our results showed that the most dominant families in the samples, including *Prevotellaceae*,* Veillonellaceae*,* Paraprevotellaceae, Lachnospiraceae, Ruminococcaceae,* and *Succinivibrionaceae* (Figure 5d). The abundances of *Prevotellaceae* in the 2.50% AMSLF and the 7.50% AMSLF groups were higher (*p *<* *0.05) than in the other groups. Furthermore, the abundance of *Veillonellaceae* was higher in the 5.00% AMSLF group, whereas, *Veillonellaceae* was lower in the 2.50% AMSLF group than in other groups. At the level of order, a total of 240 orders were detected across the treatments. *Bacteroidales*,* Clostridiales*,* Aeromonadales,* and *Spirochaetales* were the dominant orders in the samples. The abundances of *Bacteroidales* tended to be slightly high in the 2.50% AMSLF group and the 7.50% AMSLF group than in the other groups, while *Clostridiales* was significantly lower (*p *<* *0.05) in the 2.50% and the 7.50% AMSLF groups than in other groups (Figure 5c). At the genus level, a total of 249 genera was detected across the treatment samples. *Prevotella, Acidaminococcus, Coprococcus, Sutterella, Anaerovibrio, Ruminococcus, Succinivibrio,* and *Phascolarctobacterium* (Figure 5e) were the dominant genera in the samples. Our results showed that the relative abundance of the main bacteria genera were significantly (*p *<* *0.05) affected by the AMSLF supplementation.

## 4 DISCUSSION

### 4.1 Diarrheal incidence

The incident of diarrhea in weaned piglets is caused by the overpopulation of coliform bacteria, and the control of its spread constitutes an effective means of reducing the diarrheal incidence (Li et al., 2011). The results of our study showed that AMSLF supplementation significantly lowered (*p *<* *0.05) diarrheal incidence in weaned piglets throughout the experimental period. The highest diarrhea occurrences were recorded in the control group, while the lowest diarrheal incidence was observed in the 7.50% AMSLF group, hence indicating that increased in AMSLF, could effectively prevent diarrhea in piglets. Similar findings were reported by Li et al. (2011) who observed a lower diarrheal incidence in early weaned piglets supplemented with *Atractylodes macrophala* Koidz polysaccharides. Astragalus plant has high bioactive compounds content that may effectively function in preventing enteric bacteria growth in the gut therefore, decreasing the diarrheal incidence in weaned piglets.

### 4.2 Growth performance

The current study revealed that the inclusion of AMSLF in pig diet increased ADFI, leading to an increase in the growth rate, consequently improving the feed‐to‐gain ratio for the entire studies. The supplementation with 5.00% AMSLF and 7.50% AMSLF significantly (*p *<* *0.05) increased the growth performance of weaning piglets. Our results are in agreement with findings by Yuan et al. (2006) who reported that Astragalus polysaccharide (APS) in the diet increased growth performance of piglets. Similarly, Wang et al. (2010) reported that highest concentration (10,000 mg/kg) of Astragalus membranaceus root powder and Astragalus polysaccharide (APS) in the diet increased growth performance of broiler chicks. In contrast, Anguita, Canibe, Pérez, and Jensen (2006) and Wellock, Fortomaris, Houdijk, Wiseman, and Kyriazakis (2008) reported that dietary fiber supplementation decreased ADFI and growth performance in piglets raised under control conditions. The AMSLF is rich in nutritional components such as proteins, vitamins, minerals, and other bioactive compounds that may improve the palatability of the diet, hence increasing pig feed consumption. The high ADFI and growth performance of piglets in this study might be due to high nutrient contents of Astragalus roots. High feed conversion ratio was recorded in piglets fed 5.00% AMSLF and 7.50% AMSLF diets. However, no influence of Astragalus was observed on growth performance of piglets fed 2.50% AMSLF level. Therefore, dietary supplementation with AMSLF at an optimum level can positively influence the growth performance of piglets.

### 4.3 Nutrient digestibility

An important indicator of the nutritional value of feed is the utilization of nutrients in the feed by animals. In this study, digestibility of CF, CP, GE and DM in the 5.00% AMSLF and 7.50% AMSLF treatment groups was significantly (*p *<* *0.05) lower than in the 2.50% AMSLF and control groups. Although digestibility of CP, CF, GE, and DM in the 2.50% treatment group tended to be high, the value was not statistically significant. These results are in agreement with findings by Jørgensen, Zhao, and Eggum (1996) who reported a low digestibility of CP, DM and GE in pigs fed a high‐fiber diets. Similarly, Dilger, Sands, Ragland, and Adeola (2004) observed decreased in digestibility of DM and the GE in pigs supplemented with high soy hull fiber. The low nutrient digestibility observed in this study could be due to the high fiber content of Astragalus membranaceus stems and leaves.

### 4.4 Immunity

Astragalus stems and leaves contain active ingredients similar to that of the roots but vary in concentrations (Xi et al., 2014), therefore, supplementation with AMSLF may enhance immunity systems in piglets. The results of this study showed that dietary supplementation with AMSLF increased the level of IL‐2 and TNF‐α in the blood of piglets. Although the results were not significantly different among treatment groups, IL‐2 level in pig blood tended to be high in AMSLF supplemented groups. These results are in agreement with findings by Hai‐jiao (2009) and Xi et al. (2014) who reported that Astragalus stems and leaves acted as an immunoenhancing agent in chicken. The antibodies IgA, IgG and IgM antibody play a crucial role in immune response of animals to disease infection. Here, we showed that supplementation with AMSLF increased levels of IgG, IgA, and IgM in piglets at the beginning, then the levels dropped towards the end of the experimental period. The highest levels of IgA, IgG, and IgM were recorded in 5.00% AMSLF group. Similar, findings showing increased in IgA, IgG, and IgM in piglets and broilers supplemented with Astragalus plants have been reported (Feng, Liu, Xu, Liu, & Lu, 2007; Ilsley, Miller, & Kamel, 2005). These results suggest that the immune response of pig against diseases can be enhanced by AMSLF supplementation.

### 4.5 pH and volatile fatty acids

Some fiber‐based substances in the diet may not be digested in the small intestine of piglets, but microbes in the hindgut can utilize these fiber‐based foodstuffs by fermentation to produce VFAs such as acetic acid, butyric acid, and propionic acid. These VFAs accounts for about 90% of the total acid production in the hindgut of monogastric animals, representing about 10%–30% of the total energy requirement of animal (Christensen, Knudsen, Wolstrup, & Jensen, 1999). In this study, piglets supplemented with 2.50% AMSLF tended to have slightly higher concentration of acetic acid, butyric acid, propionic acid, and total VFA. These results are consistent with findings by Bikker et al. (2006) who reported that feeding high‐fermented carbohydrate diets to newly weaned piglets amplified the levels of total VFA, acetic acid and butyric acid. In contrast, Freire et al. (2000) found that the VFA level in the cecum of weaning piglets decreased at a ratio of 1.16 mg/g after changing the diet from sugar‐beet pulp to soya‐bean‐hulls. The ceca pH was slightly lower in the 2.50% AMSLF group at the beginning, then increased towards the end of the experiment, but there was no significant difference between the treatment groups. Similarly, Namkung, Li, Yu, Cottrill, and De Lange (2004) observed no effect of herbal feed and organic acids on fecal and ilea pH content of newly‐weaned pigs. Generally, optimum pH and VFA levels in cecum contents are indicators of intestinal health and microbial activity (Bindelle et al., 2009).

### 4.6 Ceca microbial composition

Animal gut serves as a habitat for many microbial organisms that may affect health, well‐being and growth performance of the host. In this study, high‐throughput 16S‐rDNA sequencing was used to describe the abundance and diversity of microorganisms in the cecum of piglets fed four different dietary treatments with AMSLF. The AMSLF regulates the final products of fermentation and the number of bacteria and bacteria diversity within the cecum due to its high fiber content. In the current study, OTU analysis showed ceca microbial diversity in all the treatment groups. The alpha diversity indices also indicated the species richness and evenness of individual sample. Furthermore, taxonomic classification of microbial composition in the ceca of piglets revealed that *Bacteroidetes, Firmicutes*,* Proteobacteria, Spirochaetes*,* Cyanobacteria,* and *Chloroflexi* were the dominant bacterial phyla, which is in agreement with the findings by Lamendella, Santo Domingo, Ghosh, Martinson, and Oerther (2011) who noted that *Firmicutes* and *Bacteroidetes* were the dominant phyla in pig fecal microbial. Our results showed a higher population of *Bacteroidetes* in the 2.5% AMSLF and the 7.5% AMSLF groups than in other treatment groups. This may be due to individual animal variation. Many factors, including age, diet, and individual animal affect gut microbial population. Regardless of dietary treatment in the current study, microbial composition of each individual piglet was unique (Figure 5a–e). These findings were supported by previous studies in which pigs fed different diets exhibited unique microbial population in their excretions as analyzed by Illumina‐based sequencing and pyrosequencing of 16SrDNA library technology (Kim et al., 2011; Pajarillo, Chae, Balolong, Kim, & Kang, 2014; Pedersen et al., 2013). At the family level, our observation revealed that *Prevotellaceae*,* Veillonellaceae*,* Paraprevotellaceae, Lachnospiraceae, Ruminococcaceae,* and *Succinivibrionaceae* were the dominant families in all samples. However, the abundance of *Veillonellaceae* and *Ruminococcaceae* tended to be lower in 2.50% AMSLF group than in other treatment groups. Recent study by Daly, Darby, and Shirazi‐Beechey (2016) revealed a significantly lower *Veillonellaceae* abundance in fecal microbes of pigs. The low abundance of *Ruminococcaceae* in the present study may be due to high VFAs concentration in the pig cecum (Cook & Russell, 1994). At the genus level, our results revealed that *Prevotella,* [*Prevotella*]*, Acidaminococcus, Coprococcus, Sutterella, Anaerovibrio, Ruminococcus, Succinivibrio,* and *Phascolarctobacterium* were the dominant genera in all samples. However, the abundance of *Prevotella* and *Ruminococcus* tended to be higher in the 2.5 AMSLF and the 7.50% AMSLF groups than in other treatment groups. Our results are in agreement with findings by Liu, Zhang, Zhang, Zhu, and Mao (2016) who reported that *Prevotella, Acetitomaculum Succiniclasticum, Saccharofermentans, Mogibacterium,* and *Ruminococcus* were the dominant genera in ruminal content and the epithelium of lactating cows. The reason for increased in abundance of *Prevotella* and *Ruminococcus* in some treatment groups is unclear. It could be concluded that supplementation with AMSLF improved growth performance and VFA production, decreased intestinal pH and enhanced health and well‐being of piglets as well as increased gut microbial population and diversity. This study establishes that inclusion of 2.50%–5.00% of AMSLF in the diet are the optimum level that improves overall performance of piglets. Therefore, it provides valuable insights into the search for the replacement of antibiotics in monogastric feeding and management.

## CONFLICT OF INTEREST

The authors declare that there is no conflict of interest.

## AUTHORS CONTRIBUTIONS

Q. G, J. H, and D. C. designed the experiment and edited the final version of the manuscript; C. W and S. A. performed the experiment; S. A and E. M. A wrote and organized the manuscript. All authors had a significant contribution to the development of the manuscript and approved the final version of the manuscript.

## DATA ACCESSIBILITY

The data will be available on request from the corresponding authors.
