# Supplementary figures and images for: Effects of Astragalus membranaceus fiber on growth performance, nutrient digestibility, microbial composition, VFA production, gut pH, and immunity of weaned pigs
Source: Microbiologyopen. 2018 Aug 16;8(5):e00712. doi: 10.1002/mbo3.712 (PMC6528644; doi:10.1002/mbo3.712)

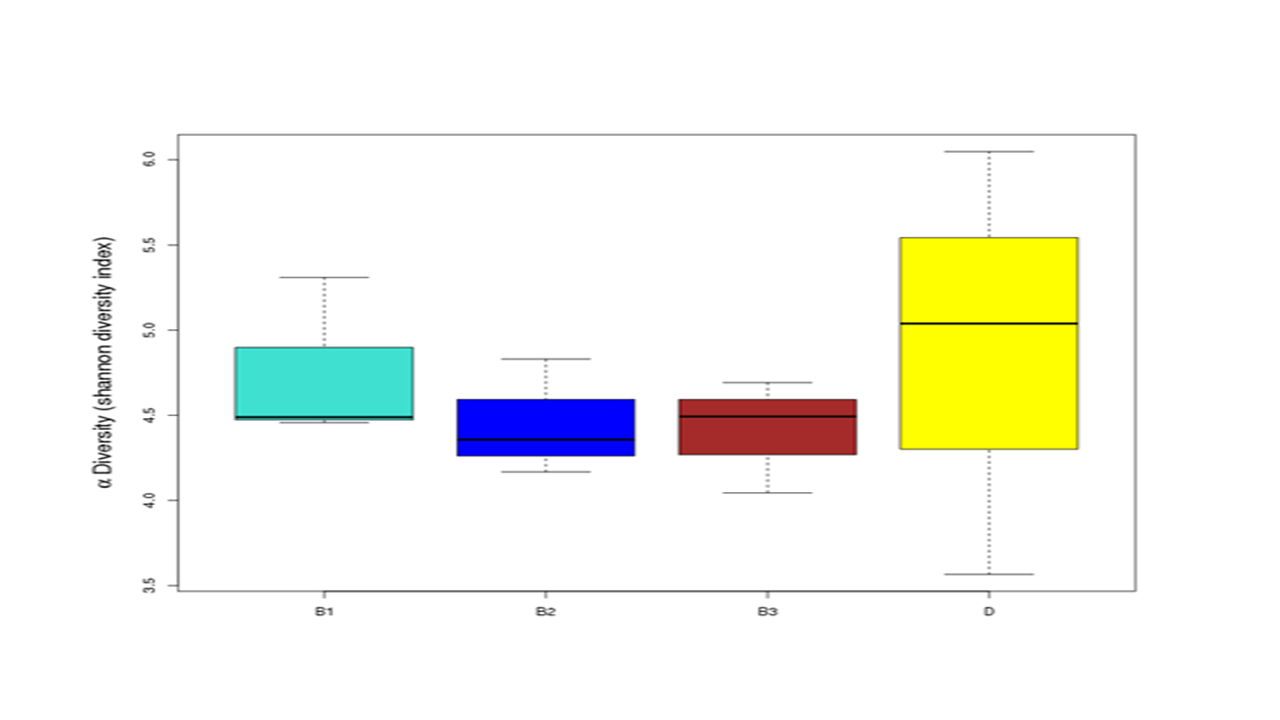

Supplement: Supplementary file 1 [file MBO3-8-e00712-s001.tif]

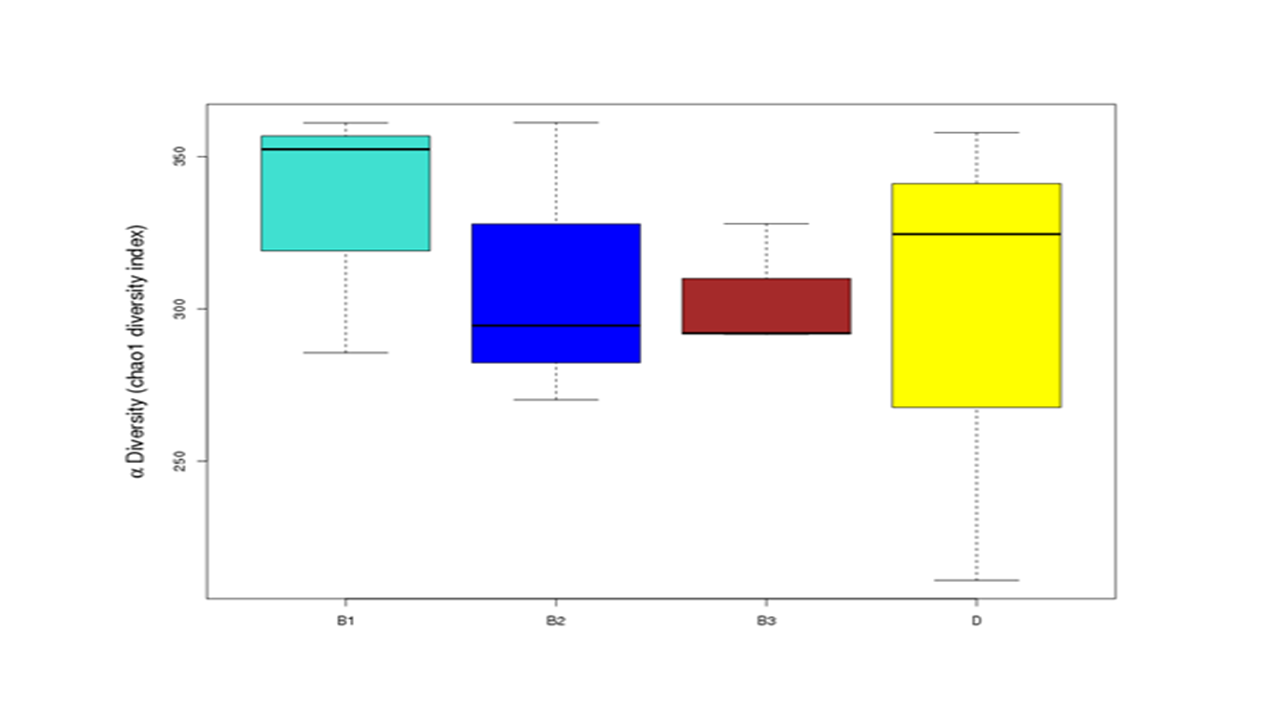

Supplement: Supplementary file 2 [file MBO3-8-e00712-s002.tif]

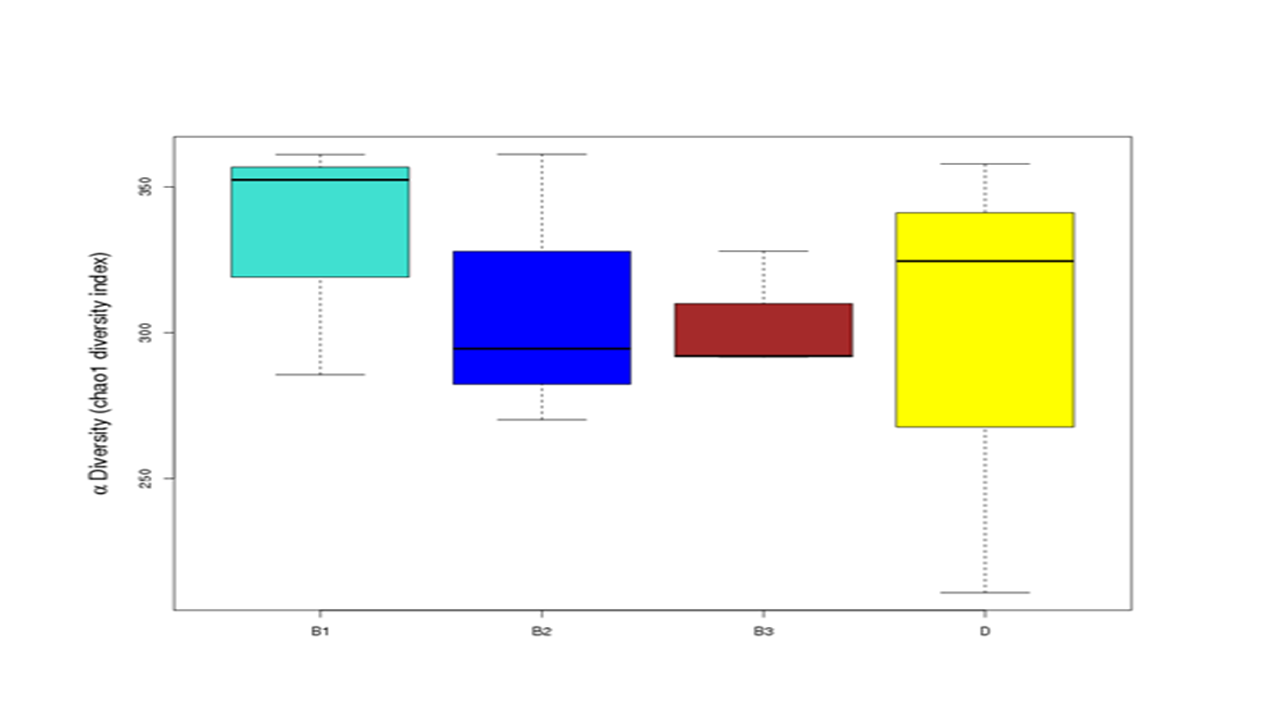

Supplement: Supplementary file 3 [file MBO3-8-e00712-s003.tif]

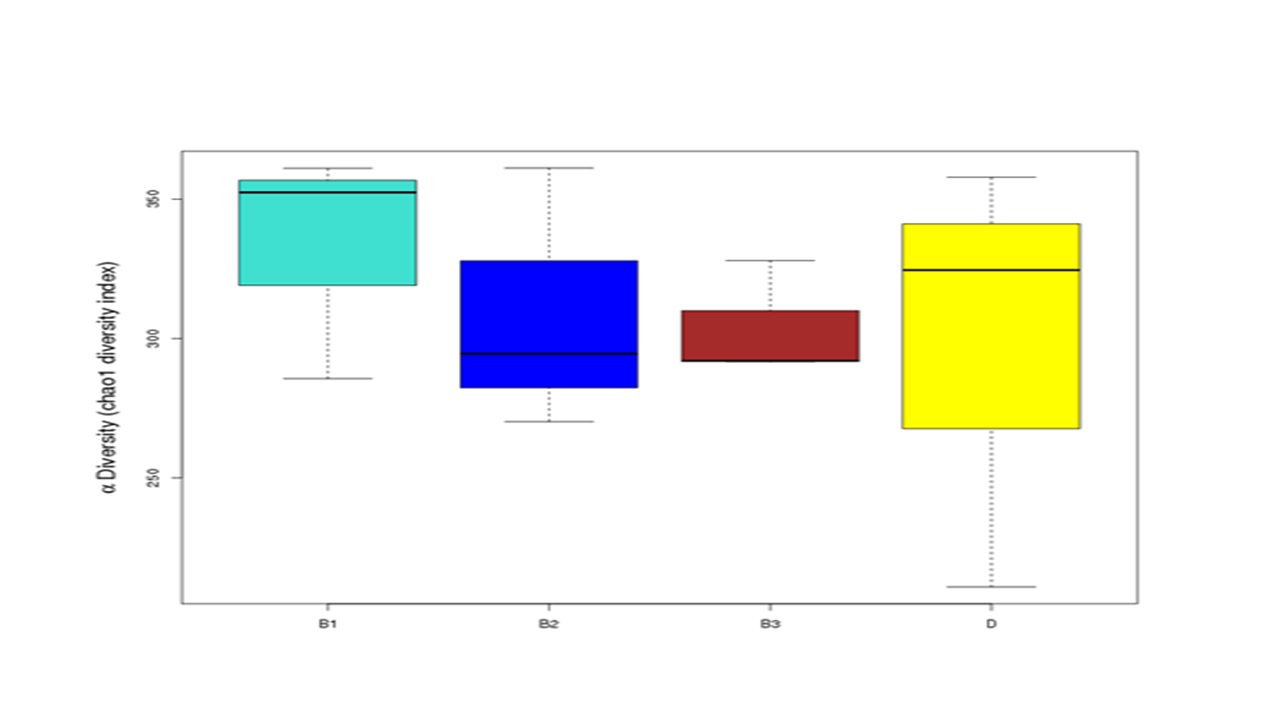

Supplement: Supplementary file 4 [file MBO3-8-e00712-s004.tif]

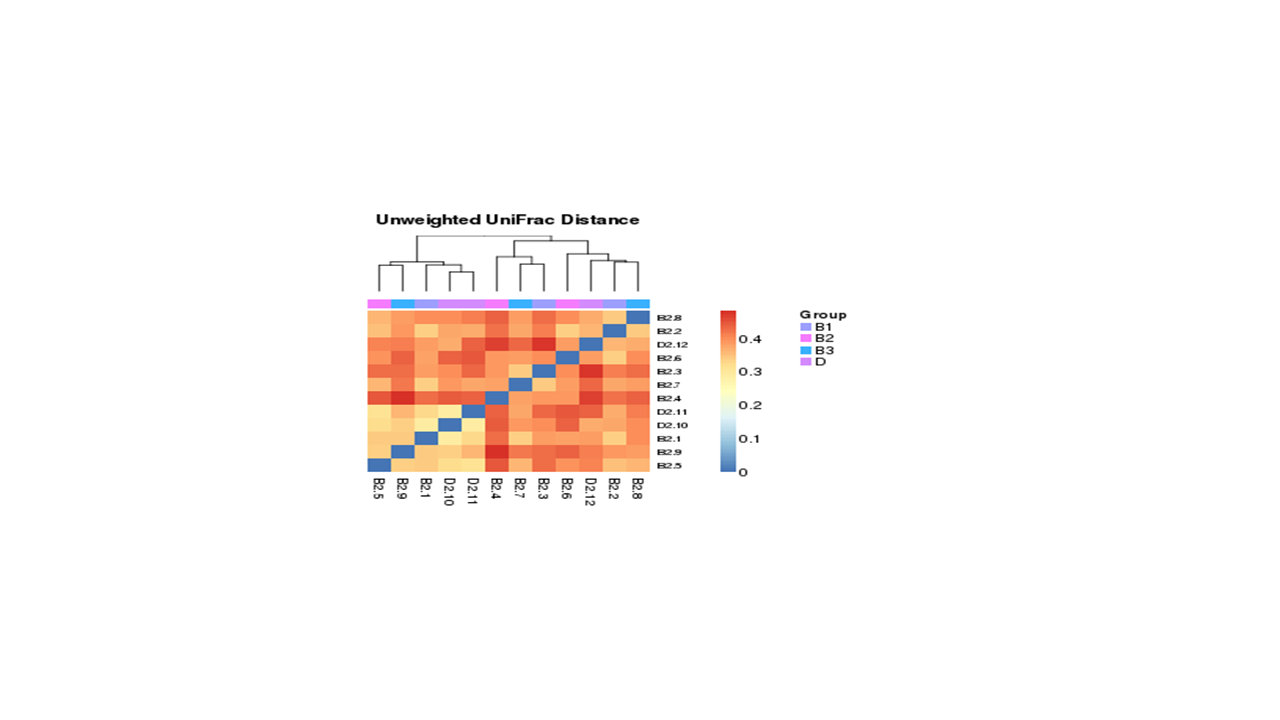

Supplement: Supplementary file 5 [file MBO3-8-e00712-s005.tif]

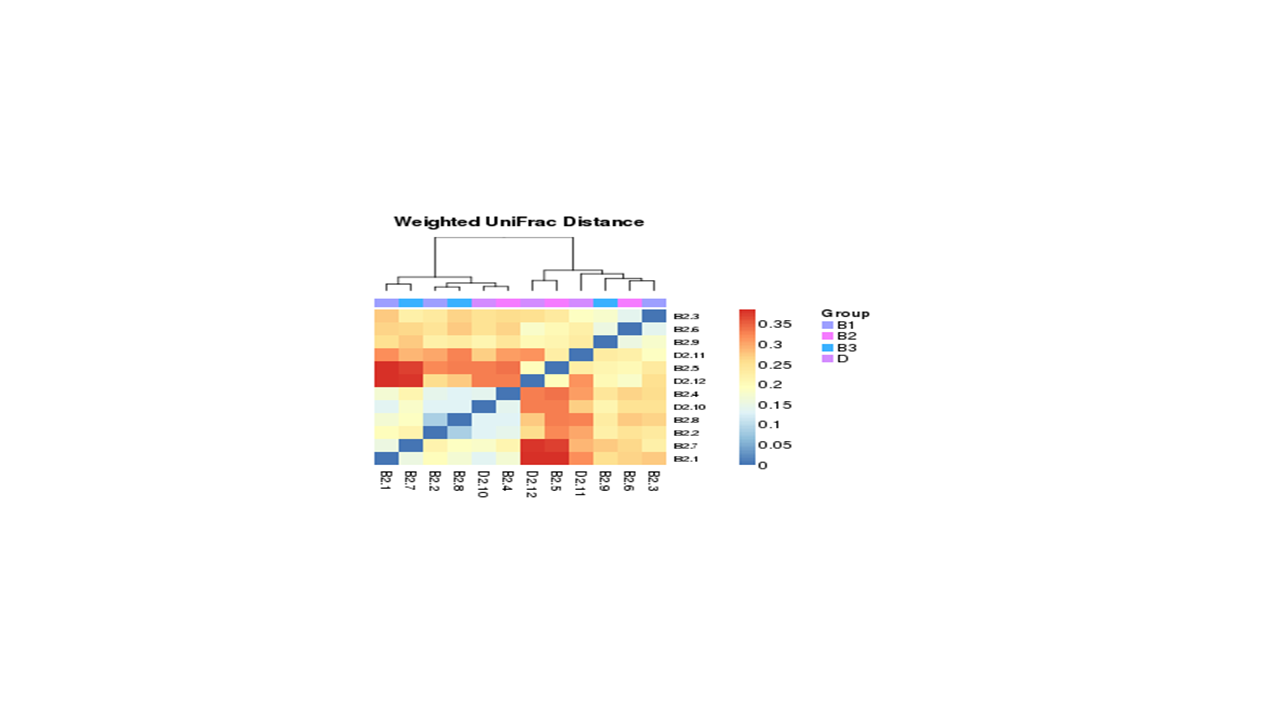

Supplement: Supplementary file 6 [file MBO3-8-e00712-s006.tif]

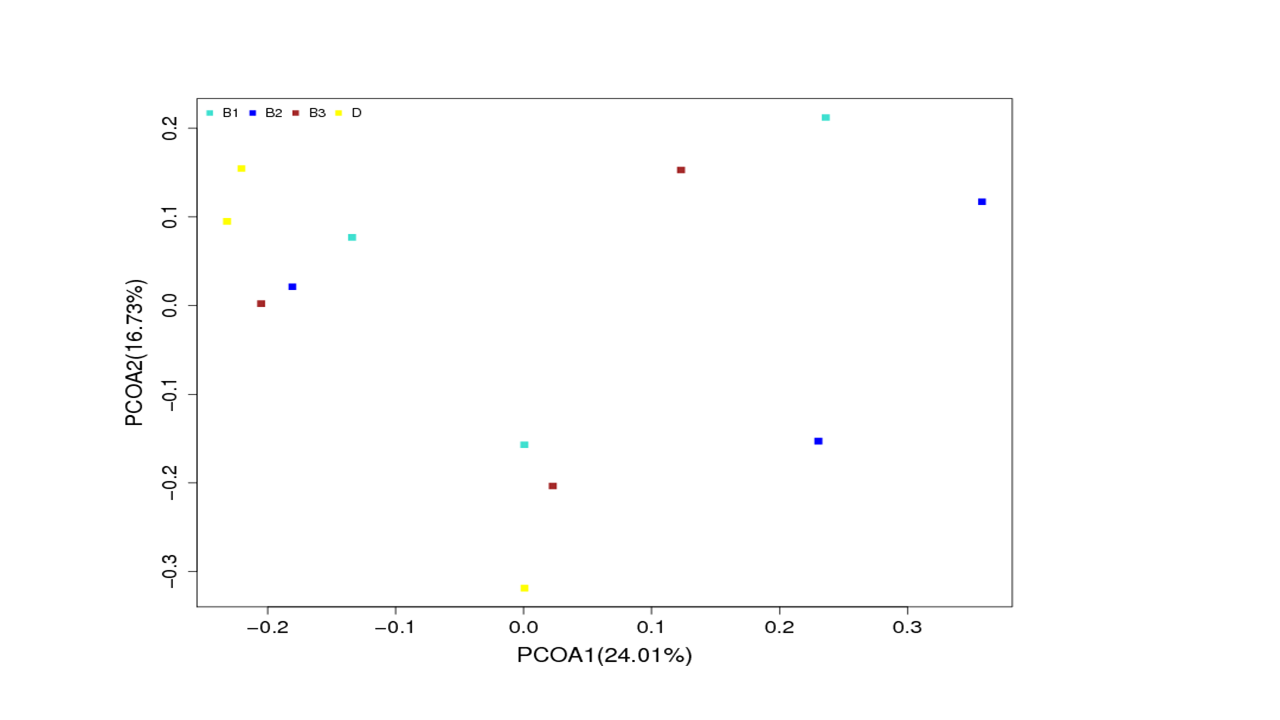

Supplement: Supplementary file 7 [file MBO3-8-e00712-s007.tif]

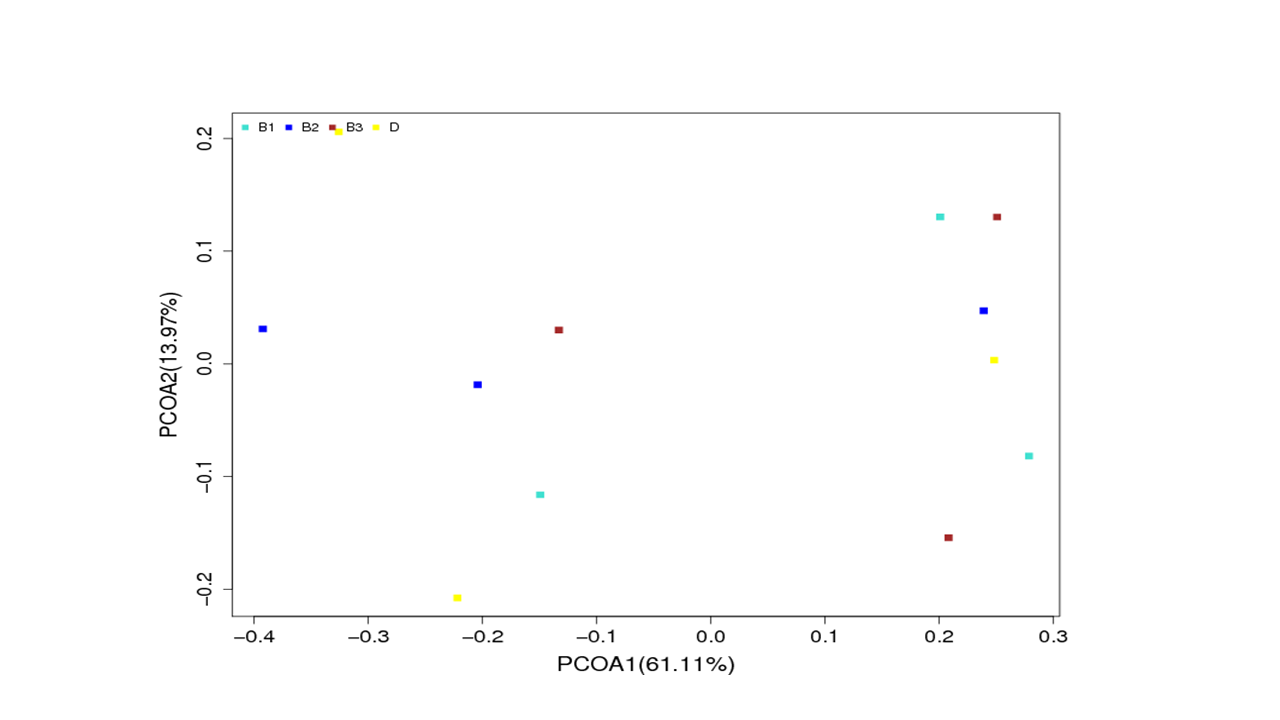

Supplement: Supplementary file 8 [file MBO3-8-e00712-s008.tif]
