# Supplementary material for: Effects of Astragalus membranaceus fiber on growth performance, nutrient digestibility, microbial composition, VFA production, gut pH, and immunity of weaned pigs
Source: Microbiologyopen. 2018 Aug 16;8(5):e00712. doi: 10.1002/mbo3.712 (PMC6528644; doi:10.1002/mbo3.712)
Supplement: Supplementary file 9 [file MBO3-8-e00712-s009.docx]

**Supplementary Materials**

**Effects of Astragalus membranaceus fiber on growth performance, nutrient digestibility, microbial composition, VFA production, gut pH and immunity of weaned pigs**

**Dongsheng Che^1, 2, 3^, Seidu Adams ^1^, Cai Wei^1, 2, 3^, Qin Gui-Xin^1, 2, 3^, Emmanuel Musa Atiba^1^, Jiang Hailong ^1, 2, 3^***

**^1^College of Animal Science and Technology, Jilin Agricultural University, Changchun 130118, China**

**^2^Key Laboratory of Animal Production, Product Quality and Security, Ministry of Education, Changchun 130118, China**

**^3^Jilin Provincial Key Laboratory of Animal Nutrition and Feed Science, Changchun 130118, China**

First Author details

**First name: Dongsheng**

**Last (Family) name: Che**

Corresponding author

**Jiang Hailong, The college of Animal Science and Technology, Jilin Agricultural University, E-mail:** [**hljiang@jlau.edu.cn**](mailto:hljiang@jlau.edu.cn)

**Seidu Adams, The college of Animal Science and Technology, Jilin Agricultural University, E-mail:** [**adamyazori@gmail.com**](mailto:adamyazori@gmail.com)

Funding information

**National Key Research and Development Program of China, Grant/Award number: 2017YFD0502104; The Scientific Project of Jilin province, Grant/Award: 20170309003NY & 20180101023JC**

**Figure S1** Shows the alpha diversity indices (wilcox.test function in R for both sets of samples and kruskal.test function in R, if more than two sets of samples were used). The Observed species index (S 1B) and the Chao index (S 1A) reflect the species richness of the communities in the sample. The Shannon index (S 1C) reflect the species diversity of the communities affected by species richness and species evenness in the sample community. The PD_whole_tree index (S 1D) reflects the difference in species preservation in the sample from evolutionary history. Group B1: 2.50% AMSLF; Group B2: 5.00% AMSLF; Group B3: 7.50% AMSLF; Group D: 0.00% AMSLF

B2: 5.00% AMSLF

B1: 2.50% AMSLF

D: 0.00% AMSLF

B3:7.50% AMSLF

**Figure S2** Shows UniFrac distance distribution heatmap of clustering samples with similar beta diversity, the clustering UniFrac results reflects the similarity between samples. The UniFrac results presented in the figure are divided into two kinds: A (unweighted UniFrac) and B (weighted UniFrac). The Weighted UniFrac considers the sequence abundance and the Unweighted UniFrac does not consider the abundance. PCoA analysis (C: Unweight_UniFrac) and (D: Weight_UniFrac). Group B1: 2.50% AMSLF; Group B2: 5.00% AMSLF; Group B3: 7.50% AMSLF; Group D: 0.00% AMSLF

D: 0.00% AMSLF

B3: 7.50% AMSLF

B2: 5.00% AMSLF

B1: 2.50% AMSLF

In the UniFrac heat maps**;**

D: 0.00% AMSLF

B3: 7.50% AMSLF

B2: 5.00% AMSLF

B1: 2.50% AMSLF

In the PCoA;
